# Markers of Glomerular and Tubular Damage in the Early Stage of Kidney Disease in Type 2 Diabetic Patients

**DOI:** 10.1155/2018/7659243

**Published:** 2018-08-09

**Authors:** Agnieszka Żyłka, Paulina Dumnicka, Beata Kuśnierz-Cabala, Agnieszka Gala-Błądzińska, Piotr Ceranowicz, Jakub Kucharz, Anna Ząbek-Adamska, Barbara Maziarz, Ryszard Drożdż, Marek Kuźniewski

**Affiliations:** ^1^St. Queen Jadwiga Clinical District Hospital No. 2, 35-301 Rzeszów, Poland; ^2^Department of Medical Diagnostics, Jagiellonian University Medical College, 30-688 Kraków, Poland; ^3^Department of Diagnostics, Chair of Clinical Biochemistry, Faculty of Medicine, Jagiellonian University Medical College, 31-501 Kraków, Poland; ^4^Faculty of Medicine, University of Rzeszów, 35-310 Rzeszów, Poland; ^5^Department of Physiology, Faculty of Medicine, Jagiellonian University Medical College, 31-531 Kraków, Poland; ^6^Department of Uro-Oncology, Maria Sklodowska-Curie Memorial Cancer Center and Institute of Oncology, 02-781 Warsaw, Poland; ^7^Diagnostic Department, University Hospital, 31-501 Kraków, Poland; ^8^Chair and Department of Nephrology, Faculty of Medicine, Jagiellonian University Medical College, 31-501 Kraków, Poland

## Abstract

Diabetic kidney disease develops in half of genetically predisposed patients with type 2 diabetes (T2DM). Early diagnosis of kidney damage and nephroprotective treatment are the ways of preventing the disease progression. Our aim was to evaluate selected laboratory markers of glomerular and tubular damage in T2DM patients with early stages of chronic kidney disease (G1/G2, A1/A2) for their associations with A2 albuminuria and early decline in the estimated glomerular filtration rate (eGFR). Among 80 T2DM patients with median eGFR of 92.4 ml/min/1.73 m^2^ and median urinary albumin to creatinine ratio (uACR) of 4.69 mg/g, 19 had uACR > 30 mg/g (A2). Higher serum cystatin C, serum and urine neutrophil gelatinase associated lipocalin (NGAL), urine kidney injury molecule 1 (KIM-1), detectable urine transferrin and IgG, and lower serum uromodulin significantly predicted A2 albuminuria, urine KIM-1/creatinine ratio, and IgG being the best predictors. Albuminuria, urine NGAL/creatinine, and IgG correlated with diabetes duration. Albuminuria, urine NGAL, transferrin, IgG, and uromodulin correlated with diabetes control. In a subgroup of 29 patients, retrospective data were available on changes in eGFR and uACR over one year. Decline in eGFR was observed in 17 patients and increase in uACR in 10 patients. Serum and urine NGAL correlated with eGFR changes. Higher urine NGAL, KIM-1/creatinine ratio, and detectable IgG were significantly associated with the increase in uACR. Widely available markers, serum cystatin C, urine IgG, transferrin, and NGAL, may help in early assessment of kidney disease in T2DM patients; however, large prospective studies are needed to confirm the conclusion.

## 1. Introduction

Diabetes is the most prevalent metabolic disease worldwide, and its complications are among the most important public health issues [1]. About 30–40% of diabetic patients, especially the genetically predisposed ones, develop diabetic kidney disease (DKD), which makes it the most frequent cause of end-stage kidney disease and renal replacement therapy [2, 3].

Early diagnosis and early initiation of nephroprotective therapy have the potential to prevent the progression of DKD toward end-stage renal disease and to improve patients' prognosis. Based on the guidelines issued by Kidney Disease Outcomes Quality Initiative (KDOQI) in 2007 [4], the repeated assessment of urine albumin/creatinine ratio (uACR) in two to three samples of morning urine together with the estimation of glomerular filtration rate (eGFR) has been recognized as the best standard screening for DKD. However, currently, it is well known that kidney damage in course of type 2 diabetes mellitus (T2DM) may occur without increased albuminuria [5]. Moreover, decrease in eGFR is not an early indicator of diabetic renal damage. Therefore, in some patients, albuminuria and eGFR are not sensitive markers of early DKD. Diagnostic imaging produces nonspecific results in DKD patients, and the thick needle biopsy is very rarely used due to invasiveness and the lack of strict clinical indications in the early stage of DKD, usually characterized by very few symptoms. Thus, there is a need to seek new biomarkers of early renal damage in patients with T2DM.

Morphological changes observed in kidneys in the course of DKD affect almost all nephron structures: glycocalyx and glomerular endothelial cells, glomerular basement membrane, podocytes and slit membranes, mesangial matrix, renal interstitium, and renal tubules [6]. To properly qualify the potential markers of renal damage in T2DM, it is useful to classify them according to the renal structure affected by the pathological process [7]. On this basis, we distinguish markers of glomerular damage including transferrin, immunoglobulin G (IgG), ceruloplasmin, type IV collagen, laminin, glycosaminoglycans, lipocalin-type prostaglandin D synthase, fibronectin, podocalyxin, vascular endothelial growth factor, cystatin C (CysC), or nephrin and markers of tubular damage such as neutrophil gelatinase-associated lipocalin (NGAL), alpha-1-microglobulin, kidney injury molecule-1 (KIM-1), N-acetyl-beta-D-glucosaminidase, angiotensinogen, uromodulin, liver-type fatty acid-binding protein (L-FABP), heart-type fatty acid-binding protein (H-FABP), the products of advanced glycation, or inflammatory markers [8]. Some biomarkers, including albumin, are filtered in the glomeruli and then reabsorbed in the proximal tubule of the nephron; increased urine excretion of such markers may indicate damage of both glomerular and tubular structures [7].

The aim of the study was the assessment of selected laboratory markers of glomerular and tubular damage in serum and urine of T2DM patients without significant decrease in the glomerular filtration rate and without significantly increased albuminuria. We evaluated the studied biomarkers as the predictors of moderately increased albuminuria and evaluated their associations with early decline of kidney function in T2DM.

## 2. Materials and Methods

### 2.1. Study Group

The cross-sectional study recruited consecutive patients with T2DM consulted at the ambulatory clinic of Nephrology Department of Clinical District Hospital No. 2 in Rzeszów, Poland, between October 2014 and November 2015. The study visit was a part of a standard care and was either arranged to assess kidney function in newly diagnosed T2DM patients or was a control visit of patients with single benign renal cysts or following treatment of urinary tract infection. A subgroup of patients attended a control visit as a part of longitudinal observation of changes in eGFR and urine albumin/creatinine ratio (uACR) [9]. Inclusion criteria were T2DM diagnosis, eGFR > 60 ml/min/1.73 m^2^, and uACR < 300 mg/g. We excluded patients diagnosed with anemia, neoplasm, connective tissue disease, infection, allergy, treated with potentially nephrotoxic drugs, and with known renal disease other than DKD. Moreover, patients with poorly controlled hypertension, decompensated heart failure, urinary tract infection, after increased physical activity, and women during menstruation as well as pregnant women were excluded to avoid nonspecific albuminuria.

Patients were subjected to detailed medical examination. Patient's history was collected focusing on the presence of comorbidities, their duration, and treatment. Weight, height, and blood pressure were measured; body mass index (BMI) was calculated, and laboratory tests were ordered. According to 2012 KDOQI guidelines [10], kidney impairment was assessed based on uACR and eGFR estimated using 2009 Chronic Kidney Disease Epidemiology Collaboration formula based on serum creatinine (CKD-EPI_Cr_). Moderately increased albuminuria (uACR between 30 and 300 mg/g or A2 albuminuria category) was considered the objective evidence of kidney disease, according to the definition of chronic kidney disease issued by Kidney Disease: Improving Global Outcomes (KDIGO) [11].

In a subgroup of patients, retrospective data were available on eGFR values and uACR changes over one-year observation. These patients were seen a year and 6 months before the study visit. During the previous visits, patients were instructed to maintain physical activity and diabetic diet. The doses of insulin or oral hypoglycemic drugs were adjusted to maintain good glycemic control. Renin-angiotensin-aldosterone system inhibitors were used as antihypertensive drugs, and statins were used in treatment of dyslipidemia if not contraindicated [12]. We assumed the decrease in eGFR based on serum creatinine and the increase in urine albumin/creatinine ratio after one year of observation as the indicators of kidney function decline [13, 14].

All patients provided informed consent for the study. The study protocol was approved by the Bioethics Committee of the Regional Medical Chamber in Rzeszów, Poland (approval number 70/2017/B issued on 19th September 2014).

### 2.2. Laboratory Tests

First morning urine and fasting blood sample were collected for laboratory measurements at the day of the study visit. The laboratory tests included routine tests used for the assessment of the patients' health status, and a set of additional tests was performed for the purpose of this study.

The routine tests included fasting serum glucose, glycated hemoglobin A_1c_ (HbA_1c_), complete blood count, lipid profile, serum C-reactive protein (CRP), and creatinine. GFR was estimated based on serum creatinine according to CKD-EPI_Cr_ formula [8]. Urinalysis with urine sediment analysis was performed using the first morning urine sample to exclude urinary tract infection. First morning urine samples were also tested for the concentration of NGAL, albumin, and creatinine, and then, uACR and urine NGAL/creatinine ratios (uNGAL/Cr) were calculated. The measurements of uNGAL were conducted using chemiluminescent microparticle immunoassay and Architect analyzer (Abbott Diagnostics, Lake Forest, IL, USA). Urine albumin concentrations were measured using an immunoturbidimetric method, and creatinine concentration was assessed using an enzymatic method on Olympus/Beckman Coulter Chemistry Analyzer AU680 (Beckman Coulter, Brea, USA). Peripheral blood counts were performed using a hematology analyzer ADVIA 2120i (Siemens Healthcare, Erlangen, Germany). Urinalysis was performed using LabUMat-UriSed 2 analyzer. The routine tests and uNGAL measurements were performed on the day of samples' collection in the Department of Diagnostics of St. Queen Jadwiga Clinical District Hospital, Rzeszów, Poland.

The remaining serum and urine were aliquoted and frozen in −80°C until the complete set of samples was collected. The tests were performed in series to avoid the cycles of repeated freezing/defreezing. The preserved material was used to determine the concentrations of transferrin, IgG, KIM-1, and uromodulin (uUMOD) in urine and NGAL, CysC, and uromodulin (sUMOD) in serum. Transferrin, IgG, and CysC were measured using an immunonephelometric method on a Nephelometer II analyzer (Siemens Healthcare, Erlangen, Germany) at the Diagnostics Department, University Hospital, Kraków, Poland. The upper reference limits were, respectively, 2.17 mg/l for urine transferrin and 3.36 mg/l for urine IgG. The reference range for serum cystatin C was 0.59–1.04 mg/l. The concentrations of sUMOD, uUMOD, serum NGAL, and urine KIM-1 were determined using commercially available enzyme-linked immunosorbent assays with a Human Uromodulin ELISA kit (BioVendor, Brno, Czech Republic), a Human Lipocalin-2/NGAL ELISA kit (BioVendor, Brno, Czech Republic), and Quantikine ELISA Human TIM-1/KIM-1/HAVCR Immunoassay (R&D Systems, McKinley Place, MN, USA), respectively. The readings were done with an automatic microplate reader Automatic Micro ELISA Reader ELX 808 (BioTek Instruments Inc., Winooski, VT, USA). The reference range for sUMOD determined by the manufacturer of the kit was 37.0–501.0 ng/ml (mean 241 ng/ml); the limit of detection was 0.12 ng/ml. The reference range for urine KIM-1 values determined by the manufacturer of the kit was 0.156–5.33 ng/ml, and mean detectable concentration was 0.009 ng/ml. The limit of detection of NGAL in serum was 0.02 ng/ml, and the reference values were 63.5 ± 33.4 for men and 64.9 ± 46.5 ng/ml for women. Uromodulin, serum NGAL, and KIM-1 measurements were performed in the Department of Diagnostics, Chair of Clinical Biochemistry, Jagiellonian University Medical College, Kraków, Poland.

### 2.3. Statistical Analysis

Data were presented as number of patients (percentage of the group) for categories, mean ± standard deviation or median (lower-upper quartile) for quantitative variables with normal and nonnormal distributions (as checked using Shapiro-Wilk's test). Spearman's rank correlation coefficient was used to study correlations. Differences between groups were tested with *t*-test and Mann–Whitney's test, according to distributions. Simple and multiple logistic regressions were used to assess predictors of G1/G2 A2 CKD. Odds ratios (OR) with 95% confidence intervals (95% CI) were reported as results of logistic regression. The variables that proved significant predictors in simple logistic regression were studied further using receiver operating characteristic (ROC) curves' analysis to assess the diagnostic accuracy. Cut-off values were chosen by maximizing the Youden index. The statistical tests were two-tailed, and the results were considered significant at *p* < 0.05. Statistica 12.0 (StatSoft, Tulsa, USA) was used for computations.

## 3. Results

### 3.1. Characteristics of Patients

The study recruited 80 patients with T2DM with median eGFR (CKD-EPI_Cr_) of 92.4 ml/min/1.73 m^2^ (79.7–101.0 ml/min/1.73 m^2^) and median uACR of 4.69 mg/g (2.86–19.17 mg/g). The patients presented with typical comorbidities, including hypertension in (67) 84%, ischemic heart disease in 10 (12%), and heart failure in 6 (7%). In most patients, diabetes was well controlled: median HbA_1c_ concentrations equaled 6.4% (5.9–7.9%). The treatment of diabetes included metformin in 74 patients (93%). Insulin therapy was necessary in 25 patients (31%).

Most patients (*N* = 61; 77%) had normal to mildly increased albuminuria (uACR < 30 mg/g). Moderately increased albuminuria (uACR between 30 and 300 mg/g) was observed in 19 patients (23%) (Table 1). On average, these patients were older and had longer known duration of diabetes, as well as higher HbA_1c_ (Table 1). Ischemic heart disease was more commonly associated with moderately increased albuminuria; however, no significant differences regarding other comorbidities nor treatment modalities were observed between patients with normal and moderately increased albuminuria (Table 1). Moreover, white blood cell counts and C-reactive protein concentrations were higher among patients with uACR between 30 and 300 mg/g (Table 1).

### 3.2. The Associations of Studied Markers with Moderately Increased Albuminuria

**Figure 1:**
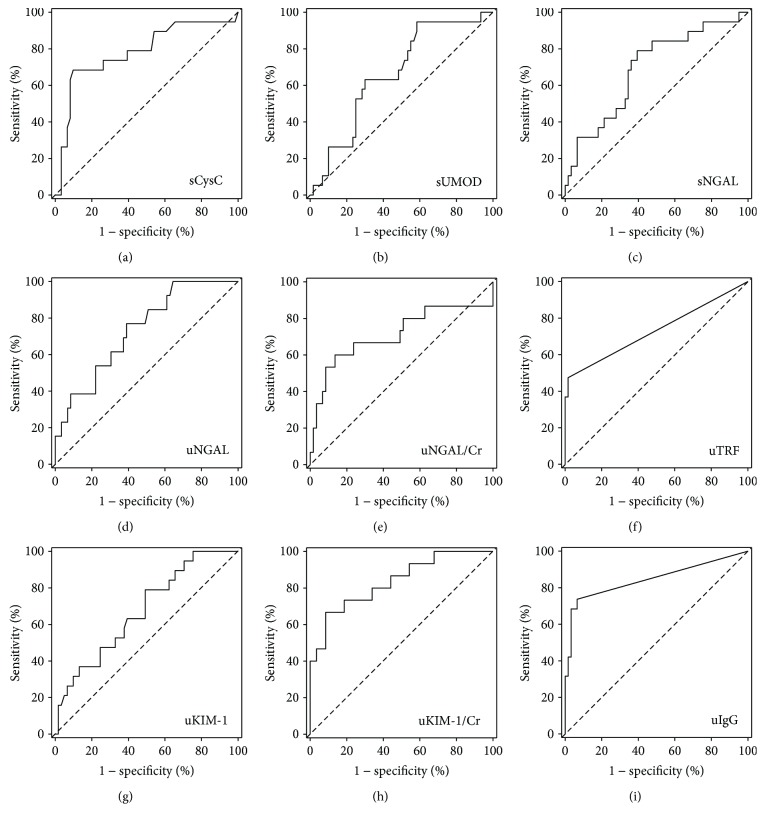
Receiver operating characteristic curves for selected serum and urine markers used to diagnose moderately increased albuminuria (uACR between 30 and 300 mg/g) among T2DM patients with eGFR > 60 ml/min/1.73 m^2^: (a) serum cystatin C (sCysC); (b) serum uromodulin (sUMOD); (c) serum NGAL (sNGAL); (d) urine NGAL (uNGAL); (e) urine NGAL/creatinine (uNGAL/Cr); (f) urine transferrin (uTRF); (g) urine KIM-1 (uKIM-1); (h) urine KIM-1/creatinine (uKIM-1/Cr); (i) urine IgG (uIgG).

Serum cystatin C, serum NGAL, urine NGAL and NGAL/creatinine ratios, urine KIM-1 and KIM-1/creatinine ratio, urine transferrin, and urine IgG were significantly higher among patients with moderately increased albuminuria while serum uromodulin was significantly lower in this group of patients (Table 1). High serum cystatin C, serum NGAL, urine NGAL and NGAL/creatinine ratio, urine KIM-1 and KIM-1/creatinine ratio, detectable urine transferrin, and urine IgG as well as low serum uromodulin were also significant predictors of moderately increased albuminuria in simple logistic regression (Table 2). Most of these predictors (except for serum concentrations of cystatin C, NGAL, and uromodulin) were independent of age, diabetes duration, the presence of hypertension, heart failure, and the treatment with renin-angiotensin-aldosterone system inhibitors (Table 2). In ROC curves' analysis, urine KIM-1/creatinine and urine IgG showed highest values of the area under the ROC curve, being the best markers to discriminate between patients with and without moderately increased albuminuria (Figure 1, Table 3). In case of most studied markers, the cut-off values selected to best discriminate between patients with normal to mildly increased and moderately increased albuminuria fell within the values observed in healthy individuals. However, this was not the case for serum cystatin C, urine transferrin, and urine IgG, where the selected cut-off values were slightly higher than the upper reference limit established by our laboratory. Among 61 patients with uACR < 30 mg/g, 11 patients (18%) had serum cystatin C above the upper reference limit of 1.04 mg/l, 4 (7%) had urine IgG above the upper reference limit of 3.36 mg/l, and 1 (2%) had urine transferrin above the upper reference limit of 2.17 mg/l.

### 3.3. Correlations of Studied Markers with eGFR, Albuminuria, Diabetes Duration, and Diabetes Control

Except for serum cystatin C, the studied serum and urine markers of renal function did not significantly correlate with eGFR based on serum creatinine (Table 4). Patients with detectable IgG and those with detectable transferrin had on average lower eGFR values (median 86 and 83 ml/min/1.73 m^2^, resp.) than those with undetectable concentrations (median 93 ml/min/1.73 m^2^ for both patients with undetectable IgG and those with undetectable transferrin); however, the differences were not statistically significant (*p* = 0.2 in case of transferrin, *p* = 0.3 in case of IgG).

Urine albumin, albumin/creatinine ratio, NGAL/creatinine ratio, and IgG significantly correlated with known duration of diabetes (Table 4). However, only serum cystatin C (*R* = 0.61; *p* < 0,001) and serum uromodulin (*R* = −0.26; *p* = 0.018) were significantly correlated with age. Moreover, urine albumin, uACR, urine NGAL and NGAL/creatinine ratio, urine transferrin, urine IgG, urine uromodulin, and uromodulin/creatinine ratio significantly correlated with diabetes control as reflected by HbA_1c_ concentrations. Urine markers NGAL, KIM-1, IgG, and transferrin were correlated with albuminuria (Table 4). Except for serum NGAL (*R* = 0.45; *p* < 0.001), the studied markers were not correlated with C-reactive protein. Also, none correlated with BMI. None of the studied markers differed significantly between men and women, between patients with and without hypertension, or between those treated and not treated with renin-angiotensin-aldosterone inhibitors.

### 3.4. Associations between Concentrations of Studied Markers and Follow-Up Data

**Figure 2:**
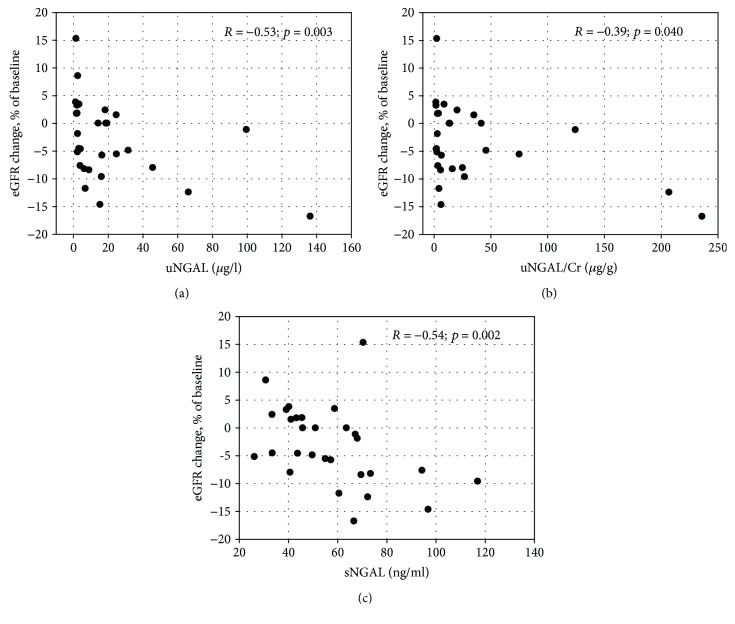
The correlations between percentage change in eGFR values over one-year observation and urine NGAL concentrations (a), urine NGAL/creatinine ratios (b), serum NGAL concentrations (c) among 29 patients for whom retrospective data were available. Change in eGFR was calculated as [(control eGFR − baseline eGFR)/baseline eGFR]∗100%. Spearman *R* coefficients and *p* values are shown on the graphs.

Among 29 patients (36% of the study group), the retrospective data were available that had been recorded one year before the study visit. The retrospective data included eGFR and uACR values. During the year, GFR decreased in 17 patients (59%) (Table 5). Median decrease equaled 5.2 ml/min/1.73 m^2^, that is, 7.6% of the initial eGFR value. Among studied markers, only higher NGAL concentrations both in serum and in urine were significantly associated with decrease in eGFR (Table 5). Moreover, the concentrations of serum NGAL, urine NGAL, and urine NGAL/creatinine ratios correlated negatively with the percentage changes in eGFR over the year (Figure 2).

**Figure 3:**
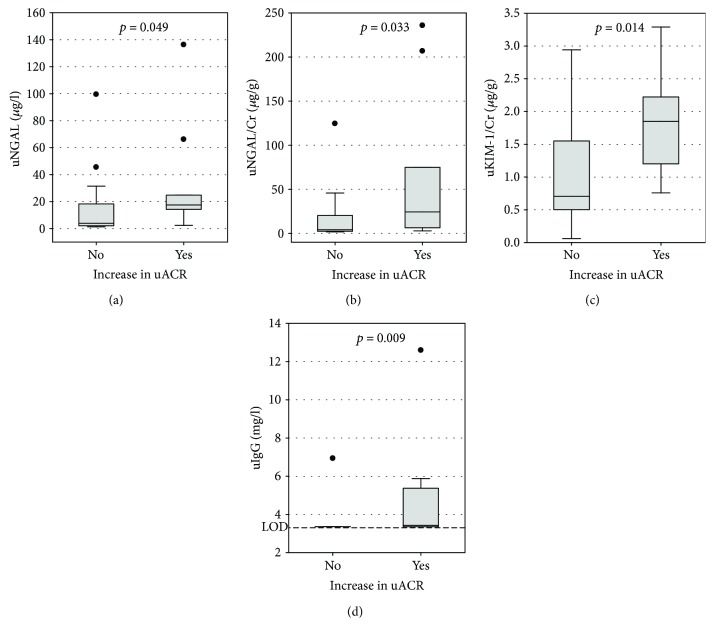
The associations between increase in urine albumin/creatinine ratio (uACR) over one-year observation and urine NGAL concentrations (a), urine NGAL/creatinine ratios (b), urine KIM-1/creatinine ratios (c), and urine IgG concentrations (d) among 29 patients for whom retrospective data were available. Data are shown as median, interquartile range (box), nonoutlier range (whiskers), and outliers (points). LOD indicates the limit of detection of urine IgG; *p* values in Mann–Whitney's test are shown on the graphs.

During the one-year observation, uACR increased in 10 patients (34%), with median increase of 12.2 mg/g. We observed no relationship between the decrease in eGFR and the increase in albuminuria (*p* = 0.9). However, higher urine NGAL, urine NGAL/creatinine ratio, urine KIM-1/creatinine ratio, and detectable urine IgG were significantly associated with the increase in uACR (Figure 3).

We compared the longitudinal data (i.e., the incidence of decrease in eGFR and increase in uACR over one-year observation) between patients with studied markers below and above the cut-off values selected in a cross-sectional study (as presented in Table 3). Serum NGAL above 61 *μ*g/l was the only marker significantly associated with more prevalent decrease in eGFR (it occurred in 53% of patients with serum NGAL above the cut-off versus 17% of those with serum NGAL below the cut-off; *p* = 0.047). However, several markers were associated with more prevalent increase in uACR, that is, urine NGAL above 14.3 *μ*g/l (70% versus 32%; *p* = 0.048), urine NGAL/creatinine above 28.3 *μ*g/g (50% versus 11%; *p* = 0.023), urine KIM-1/creatinine above 1.81 *μ*g/g (50% versus 11%; *p* = 0.018), and urine IgG above 3.49 mg/l (50% versus 5%; *p* = 0.005).

## 4. Discussion

Based on KDOQI definitions [4, 10], DKD diagnosis is based on increased albuminuria and decreased eGFR. However, there are several weaknesses of albuminuria as a marker of (early) DKD. Most importantly, among T2DM who develop kidney disease associated with decrease in glomerular filtration, there are about 30–45% in whom no increased albuminuria (of above 30 mg/g creatinine) is observed [15, 16]. Further, albuminuria is not specific for DKD, and the comorbidities often observed in T2DM (such as hypertension or obesity) may also affect the filtration barrier of the glomeruli leading to increased albuminuria [17]. Moreover, treatment of hypertension often includes renin-angiotensin-aldosterone inhibitors (angiotensin-converting enzyme inhibitors and angiotensin receptor blockers) that lower hydrostatic pressure in glomeruli and thus normalize albuminuria. Although beneficial, this effect influences the diagnosis of DKD based on current guidelines [10]. There is an ongoing debate on lowering the cut-off value for albuminuria; evidence exists that the urine albumin/creatinine ratio exceeding 15 mg/g should be used to define chronic kidney disease as it better predicts kidney disease-related cardiovascular complications [18]. On the other hand, decreased eGFR occurs rather late following changes in the kidney in DKD, the early damage being often accompanied by hyperfiltration [19]. Both routine markers of DKD (albuminuria and eGFR) are altered in consequence of the damage to renal glomeruli. However, in about 30% of T2DM patients with kidney impairment, changes in renal interstitium and tubules precede damage of glomeruli [20]. Nevertheless, markers of tubular damage are currently not a part of routine assessment of T2DM patients. Our study is a part of extensive efforts aiming at the evaluation of other urinary and serum markers for their possible usefulness in early diagnosis of DKD. However, we used eGFR and albuminuria as the standard indicators of renal function at baseline and after one year of follow-up.

Our study recruited patients without significant decrease in eGFR and without severely increased albuminuria. According to currently accepted definitions [11], in individuals with eGFR > 60 ml/min/1.73 m^2^, moderately increased albuminuria serves as the evidence of stage A2 chronic kidney disease. In our study, patients with A2 albuminuria presented higher concentrations of most studied markers of both glomerular (serum cystatin C, urine IgG, and transferrin) and tubular damage (urine and serum NGAL, urine KIM-1), as well as lower serum concentrations of uromodulin. A2 patients were older, with longer history of diabetes, and there was a positive correlation between albuminuria and diabetes control, similar to previous reports [21]. Still, in case of urine IgG, transferrin, NGAL, and KIM-1, the difference between A1 and A2 subgroups remained significant after adjustment for age, diabetes duration, and comorbidities (hypertension and heart failure) as well as treatment with renin-angiotensin-aldosterone system inhibitors.

Using ROC curves analysis, we determined the cut-off values that best discriminate between A1 and A2 patients. The cut-off values for studied markers of glomerular damage, that is, serum cystatin C, urine IgG, and transferrin were higher as compared with upper reference limits established in healthy individuals. A substantial proportion (18%) of G1-G2/A1 patients presented with serum cystatin C above the upper reference limit, in line with previous reports [22, 23]. In our study, neither of the tubular markers was significantly correlated with eGFR based on serum creatinine. Moreover, serum creatinine and eGFR based on creatinine did not differ between our A1 and A2 patients, while serum cystatin C and eGFR based on cystatin C differed significantly. The KDIGO guidelines [11] recommend the estimation of GFR based on serum cystatin C in adult patients with suspected stage G3a of chronic kidney disease (GFR between 45 and 60 ml/min/1.73 m^2^). Our results suggest that measurements of serum cystatin C accompanied by calculation of eGFR with the use of CKD-EPI_CycC_ equation may be useful already at the earlier stages of diabetic kidney disease. The recently performed laboratory standardization of cystatin C measurements is also the advantage that must be remembered.

Urine IgG and transferrin excretion has been previously observed in normoalbuminuric patients with diabetes and has been associated with hypertension and hyperfiltration [24, 25]. In accordance with previous studies [26, 27], we observed increased urine IgG and transferrin in a subgroup with moderately increased albuminuria and both proteins were specific markers of kidney disease. These are notable observations, especially considering high molecular weight of IgG (150 kDa) that preserves the protein from being filtered by healthy glomeruli [28].

In our study, both urine NGAL and KIM-1 correlated positively with albuminuria, consistently with previous reports regarding patients with T2DM [29–31] and were significantly higher in patients with moderately increased albuminuria. Nauta et al. [32] observed higher concentrations of urine NGAL and KIM-1 in normoalbuminuric diabetic patients comparing to healthy subjects. Also, we have previously reported increased urine NGAL concentrations among patients with early stages or risk for DKD [33], similarly to other groups [34, 35].

Tamm-Horsfall's protein or uromodulin is the main protein present in normal urine. This glycoprotein is exclusively expressed in the thick ascending limb of the loop of Henle and proximal part of the distal renal tubule and is released to urine by partial proteolysis [36]. It was demonstrated that the damage of renal tubules in chronic kidney disease (including early stages: G1 and G2) was associated with reduced concentrations of uromodulin in both urine and serum [37–39], which reflects a pathophysiological difference of this glycoprotein compared to other renal tubule injury markers. In our study, neither serum nor urine concentrations of uromodulin were correlated with albuminuria. Moreover, we have not observed correlations between uromodulin and eGFR. However, an interesting observation is negative correlation between HbA_1c_ and urine uromodulin, suggesting tubular impairment among patients with worse glycemic control. Previously, Leiherer et al. [40] demonstrated decreased concentrations of uromodulin in urine of patients with impaired carbohydrate metabolism, including ones with T2DM.

From practical point of view, it is important for clinical practitioners that both glomerular (albumin, uACR) and tubular (urine NGAL, transferrin, IgG, and uromodulin) markers positively correlated with glycemic control, as assessed with HbA_1c_ measurements. Hence, good glycemic control is important both for the glomeruli and tubules in the early stages of diabetic nephropathy.

In a subgroup of patients, we were able to obtain longitudinal data on changes in standard measures of renal function (eGFR and uACR) over one-year observation. During the year, patients were treated according to current recommendations [12]. The concentrations of NGAL in both serum and urine as well as urine NGAL/creatinine ratios were correlated with decline in eGFR. We did not observe significant correlations between eGFR changes and other studied glomerular or tubular biomarkers. This may be caused by the small number of patients in the longitudinally observed subgroup, as well as by the relatively short period of observation [14]. However, we were able to show significant associations between the concentrations of KIM-1, NGAL, and IgG in urine and the increase in uACR in longitudinal observation. Urine KIM-1/creatinine ratio and NGAL/creatinine ratio were also examined by Nowak et al. [41] who studied the association of the markers with decline in kidney function in T2DM patients over a follow-up period of 5–12 years. Both markers were significantly associated with renal decline in simple analysis; however, in case of the NGAL/creatinine ratio, the association was weak and only KIM-1 remained a significant predictor in multiple analysis. To the contrary, we have not observed the correlation between urine KIM-1 or KIM-1/creatinine ratio and eGFR decline, although it correlated with the increase in albuminuria.

Our study, in accordance to previous ones, suggests that various pathways may be involved in early renal damage among T2DM patients [35, 41, 42]. Although albuminuria remains one of the most significant predictors of renal decline [42], other markers, such as serum and urine NGAL, serum cystatin C, urine KIM-1, IgG, and transferrin, may add to the early diagnosis of diabetic kidney disease.

The main limitations of our study are the cross-sectional setting and relatively low number of patients. In a subgroup of patients, we were able to obtain the longitudinal data on changes in standard measures of renal function (eGFR, uACR); however, these are retrospective data, and the number of patients is small. In this subgroup of patients, the studied markers were measured at the end of the follow-up, so we were only able to evaluate the associations between the studied markers and the changes in renal function during the preceding year. The diagnosis of kidney disease was not confirmed by renal biopsy, as our patients did not have clinical indications for biopsy. Therefore, we adopted surrogate markers of kidney disease, that is, the presence of moderately increased albuminuria, decrease in eGFR, and increase in uACR. Also, lack of the control group studied simultaneously with the patients' group limits our findings; however, we relied on previous experience of our laboratory with most studied markers.

In summary, the recruitment of the uniform group of T2DM patients with eGFR > 60 ml/min/1.73 m^2^ and uACR < 300 mg/g, with exclusion of important interfering conditions, enabled us to study the concentrations of selected glomerular and tubular markers in early stages of DKD. In most of our patients, the diabetes duration was below 10 years and the diabetes was well controlled. Both glomerular (serum cystatin C, urine IgG, and transferrin) and tubular (urine NGAL, KIM-1 and uromodulin) markers differed significantly between patients with moderately increased albuminuria comparing to those with normal to mildly increased albuminuria. Serum and urine NGAL were significantly associated with eGFR decline. Our findings indicate that the widely available markers such as serum cystatin C, urine IgG, transferrin, and NGAL may help in early assessment of kidney disease in T2DM patients, although large prospective studies are needed to confirm the conclusion.

## Figures and Tables

**Table 1:** Clinical characteristics and the results of laboratory tests of T2DM patients with eGFR > 60 ml/min/1.73 m^2^ according to albuminuria categories.

Characteristic	Normal to mildly increased albuminuria: uACR < 30 mg/g (*N* = 61)	Moderately increased albuminuria: uACR 30–300 mg/g (*N* = 19)	*p* value
Age, years	59 ± 11	67 ± 12	0.007^#^
Male sex, *N* (%)	32 (52)	6 (32)	0.1
Known diabetes duration, years	5 (2–10)	10 (6–15)	0.009^#^
Hypertension, *N* (%)	50 (82)	17 (89)	0.4
Ischemic heart disease, *N* (%)	5 (8)	5 (26)	0.037^#^
Heart failure, *N* (%)	3 (5)	3 (16)	0.1
BMI, kg/m^2^	31.5 ± 5.0	31.9 ± 7.5	0.8
Insulin treatment, *N* (%)	17 (28)	8 (42)	0.2
Statin use, *N* (%)	27 (44)	13 (68)	0.07
ACEI/ARB use, *N* (%)	40 (66)	13 (68)	0.8
Hemoglobin, g/dl	14.0 ± 1.4	13.2 ± 1.7	0.045^#^
White blood cell count, ×10^3^/*μ*l	7.20 (6.20–8.33)	8.51 (6.65–10.30)	0.039^#^
Fasting glucose, mmol/l	6.95 ± 0.84	7.25 ± 0.70	0.2
HbA_1c_, %	6.30 (5.90–7.80)	7.35 (6.30–8.40)	0.049^#^
Total cholesterol, mmol/l	4.68 (3.85–5.74)	4.84 (3.98–5.72)	0.8
HDL-cholesterol, mmol/l	1.19 (1.06–1.42)	1.22 (1.01–1.40)	0.7
LDL-cholesterol, mmol/l	2.66 (1.86–3.49)	2.49 (1.94–3.54)	0.8
Triglycerides, mmol/l	1.53 (1.15–2.03)	1.83 (1.40–2.80)	0.07
C-reactive protein, mg/l	2.80 (1.30–5.80)	6.90 (3.40–14.60)	0.008^#^
Serum creatinine, *μ*mol/l	68.1 (59.2–78.7)	64.5 (53.0–79.6)	0.6
eGFR (CKD-EPI_Cr_), ml/min/1.73 m^2^	94 (81–101)	86 (72–97)	0.2
Serum cystatin C, mg/l	0.86 (0.78–1.01)	1.15 (0.93–1.37)	<0.001^#^
eGFR (CKD-EPI_CysC_), ml/min/1.73 m^2^	93 (78–103)	71 (62–95)	0.002^#^
Serum NGAL, *μ*g/l	53.8 (43.3–70.4)	67.2 (61.0–103.1)	0.013^#^
Urine NGAL, *μ*g/l	10.3 (3.1–21.8)	24.7 (14.3–43.9)	0.008^#^
Urine NGAL/creatinine, *μ*g/g	9.02 (2.97–18.84)	35.1 (8.89–74.92)	0.012^#^
Urine KIM-1, *μ*g/l	0.73 (0.32–1.54)	1.26 (0.73–2.92)	0.022^#^
Urine KIM-1/creatinine, *μ*g/g	0.98 (0.30–1.31)	1.91 (1.20–3.29)	<0.001^#^
Urine transferrin, mg/l	<2.17	<2.17 (<2.17–6.43)	<0.001^#^
Detectable urine transferrin (≥2.17 mg/l), *N* (%)	1 (2)	9 (47)	<0.001^#^
Urine IgG, mg/l	<3.36	5.88 (<3.36–12.60)	<0.001^#^
Detectable urine IgG (≥3.36 mg/l), *N* (%)	4 (7)	14 (74)	<0.001^#^
Serum uromodulin, *μ*g/l	127 (95–173)	100 (58–138)	0.031^#^
Urine uromodulin, mg/l	6.56 (2.19–14.38)	5.60 (2.09–13.41)	0.5
Urine uromodulin/creatinine, mg/g	7.62 (2.18–15.95)	9.19 (3.31–12.79)	0.8

^#^Statistically significant difference between the groups. Abbreviations: T2DM: type 2 diabetes mellitus; uACR: urine albumin to creatinine ratio; BMI: body mass index; ACEI: angiotensin-converting enzyme inhibitor; ARB: angiotensin receptor blocker; HbA_1c_: glycated hemoglobin A1c; HDL: high-density lipoprotein; LDL: low-density lipoprotein; eGFR: estimated glomerular filtration rate; CKD-EPI: Chronic Kidney Disease Epidemiology Collaboration; Cr: creatinine; CysC: cystatin C; NGAL: neutrophil gelatinase-associated lipocalin; KIM-1: kidney injury molecule-1; IgG: immunoglobulin G.

**Table 2:** Odds ratios for moderately increased albuminuria (uACR between 30 and 300 mg/g) among T2DM patients with eGFR > 60 ml/min/1.73 m^2^ in simple and multiple logistic regressions adjusted for age, diabetes duration, the presence of hypertension, heart failure, and the treatment with renin-angiotensin-aldosterone system inhibitors.

Predictor variable	Simple analysis		Multiple analysis	
	Odds ratio (95% confidence interval)	*p* value	Odds ratio (95% confidence interval)	*p* value
Serum creatinine, per 1 *μ*mol/l	0.99 (0.96–1.02)	0.6	0.98 (0.94–1.01)	0.2
Serum cystatin C, per 1 mg/l	33.09 (2.82–387.83)	0.005^#^	14.98 (0.64–353.13)	0.09
Serum NGAL, per 1 *μ*g/l	1.02 (1.00-1.04)	0.018^#^	1.02 (1.00-1.04)	0.059
Urine NGAL, per 1 *μ*g/l	1.04 (1.01–1.07)	0.016^#^	1.04 (1.00-1.07)	0.035^#^
Urine NGAL/creatinine, per 1 *μ*g/g	1.02 (1.00-1.035)	0.018^#^	1.02 (1.00-1.04)	0.035^#^
Urine KIM-1, per 1 *μ*g/l	1.64 (1.09–2.45)	0.020^#^	1.87 (1.11–3.15)	0.016^#^
Urine KIM-1/creatinine, per 1 *μ*g/g	5.63 (2.16–14.68)	<0.001^#^	7.12 (2.22–22.87)	<0.001^#^
Detectable urine transferrin	54.00 (5.95–490.37)	<0.001^#^	54.90 (4.70–640.90)	0.001^#^
Detectable urine IgG	39.90 (9.25–172.10)	<0.001^#^	59.37 (8.54–412.79)	<0.001^#^
Serum uromodulin, per 1 *μ*g/l	0.99 (0.98–1.00)	0.049^#^	0.99 (0.98–1.00)	0.09
Urine uromodulin, per 1 mg/l	0.96 (0.89–1.02)	0.2	0.95 (0.88–1.03)	0.2
Urine uromodulin/creatinine, per 1 mg/g	1.00 (0.95–1.06)	0.9	1.01 (0.95–1.07)	0.8

^#^Statistically significant result. Abbreviations: see Table 1.

**Table 3:** Diagnostic accuracy data for selected serum and urine markers used to diagnose moderately increased albuminuria (uACR between 30 and 300 mg/g) among T2DM patients with eGFR > 60 ml/min/1.73 m^2^. Values observed in healthy individuals are shown to enable comparison with selected cut-off values.

Marker	Reference values previously associated with healthy individuals	Detection of moderately increased albuminuria in T2DM			
		AUC (95% CI)	Selected cut-off value	Sensitivity, %	Specificity, %
Serum cystatin C, mg/l	0.59–1.04^a^	0.78 (0.65–0.91)	1.09	68	90
Serum NGAL, *μ*g/l	Men 63.5 ± 33.4 Women 64.9 ± 46.5^b^	0.69 (0.56–0.83)	61.0	79	61
Urine NGAL, *μ*g/l	10.9 (6.0–38.2)^c^	0.74 (0.60–0.87)	14.3	80	61
Urine NGAL/creatinine, *μ*g/g	12.2 (5.9–27.9)^c^	0.71 (0.53–0.89)	28.3	60	87
Urine KIM-1, *μ*g/l	0.156–5.33^b^	0.68 (0.54–0.81)	0.73	79	51
Urine KIM-1/creatinine, *μ*g/g	0.225–3.20^b^	0.84 (0.72–0.95)	1.81	67	92
Urine transferrin, mg/l	<2.17^a^	0.73 (0.58–0.88)	2.41	47	98
Urine IgG, mg/l	<3.36^a^	0.85 (0.72–0.97)	3.49	74	93
Serum uromodulin, *μ*g/l^∗^	191.2 (89.1–299.1)^c^	0.66 (0.53–0.80)	144	95	43

^a^Reference interval used in the laboratory that performed the measurement for the present study. ^b^Reference values reported by the manufacturer of the test used in the present study [43–45]. ^c^Previously reported values measured in the same laboratory and with the same tests as in the present study: urine NGAL, urine NGAL/creatinine [46], and serum uromodulin [38]. ^∗^Low concentrations are associated with renal impairment. Abbreviations: see Table 1.

**Table 4:** Correlations between studied serum and urine markers and eGFR, diabetes duration, and HbA_1c_ concentrations.

Marker	eGFR (CKD-EPI_Cr_)		Diabetes duration		HbA_1c_		uACR	
	*R*	*p* value	*R*	*p* value	*R*	*p* value	*R*	*p* value
Serum cystatin C	−0.72	<0.001^#^	0.17	0.1	−0.05	0.7	0.21	0.08
Urine albumin	0.02	0.9	0.21	0.071	0.28	0.020^#^	NA	
Urine albumin/creatinine	−0.01	0.9	0.25	0.037^#^	0.29	0.015^#^	NA	
Serum NGAL	−0.15	0.2	0.09	0.4	0.10	0.4	0.13	0.3
Urine NGAL	−0.06	0.6	0.19	0.1	0.24	0.048^#^	0.39	<0.001^#^
Urine NGAL/creatinine	−0.05	0.7	0.24	0.046^#^	0.25	0.035^#^	0.36	0.002^#^
Urine KIM-1	0.11	0.4	−0.03	0.8	0.08	0.5	0.32	0.005^#^
Urine KIM-1/creatinine	0.05	0.7	0.05	0.7	0.16	0.2	0.45	<0.001^#^
Urine transferrin	−0.17	0.1	0.19	0.1	0.32	0.006^#^	0.48	<0.001^#^
Urine IgG	−0.14	0.2	0.27	0.016^#^	0.33	0.005^#^	0.61	<0.001^#^
Serum uromodulin	0.16	0.2	0.06	0.6	0.03	0.8	−0.09	0.4
Urine uromodulin	0.08	0.5	−0.14	0.2	−0.35	0.002^#^	−0.03	0.8
Urine uromodulin/creatinine	0.04	0.7	−0.12	0.3	−0.32	0.009^#^	0.01	0.9

^#^Statistically significant correlation. Abbreviations: see Table 1; NA: not applicable.

**Table 5:** Characteristics of 29 patients for whom retrospective longitudinal data were available. The group was divided according to changes in eGFR over one-year observation.

Characteristic	Patients with decrease in eGFR (*N* = 17)	Patients without decrease in eGFR (*N* = 12)	*p* value
Age, years	64 ± 15	63 ± 11	0.4
Male sex, *N* (%)	11 (65)	6 (50)	0.4
Known diabetes duration, years	7 (2–11)	10 (4–11)	0.4
HbA_1c_, %	6.10 (5.70–6.45)	6.20 (5.70–7.00)	0.7
Serum creatinine, *μ*mol/l	61.9 (59.2–72.5)	68.9 (61.4–77.8)	0.5
eGFR (CKD-EPI_Cr_), ml/min/1.73 m^2^	91 (81–98)	96 (80–99)	0.8
Serum cystatin C, mg/l	0.92 (0.82–1.14)	0.86 (0.76–1.10)	0.2
eGFR (CKD-EPI_CysC_), ml/min/1.73 m^2^	87 (67–96)	96 (70–105)	0.2
Urine albumin, mg/l	8.06 (6.76–13.53)	10.18 (6.21–14.65)	0.9
Urine albumin/creatinine, mg/g	7.93 (3.38–13.38)	8.07 (5.50–16.4)	0.6
Serum NGAL, *μ*g/l	66.7 (49.7–72.3)	44.4 (39.7–55.0)	0.028^#^
Urine NGAL, *μ*g/l	15.3 (4.0–31.5)	2.9 (1.9–18.5)	0.048^#^
Urine NGAL/creatinine, *μ*g/g	6.47 (3.36–45.78)	8.82 (2.42–20.3)	0.3
Urine KIM-1, *μ*g/l	1.05 (0.42–1.64)	1.12 (0.35–1.69)	0.9
Urine KIM-1/creatinine, *μ*g/g	1.01 (0.55–1.58)	1.34 (0.59–2.23)	0.5
Urine transferrin, mg/l	<2.17	<2.17	0.1
Detectable urine transferrin (≥2.17 mg/l), *N* (%)	0	2 (17)	0.08
Urine IgG, mg/l	<3.36	<3.36 (<3.36–4.00)	0.2
Detectable urine IgG (≥3.36 mg/l), *N* (%)	2 (12)	4 (33)	0.2
Serum uromodulin, *μ*g/l	125 (97–142)	105 (61–144)	0.5
Urine uromodulin, mg/l	11.30 (6.38–15.50)	4.94 (2.99–14.41)	0.3
Urine uromodulin/creatinine, mg/g	11.20 (6.67–23.17)	10.07 (4.51–15.34)	0.5

^#^Statistically significant difference between the groups. Abbreviations: see Table 1.

## Data Availability

The data used to support the findings of this study are available from the corresponding author upon request.
